# Could a B-1 Cell Derived Phagocyte “Be One” of the Peritoneal Macrophages during LPS-Driven Inflammation?

**DOI:** 10.1371/journal.pone.0034570

**Published:** 2012-03-30

**Authors:** Ana Flavia Popi, Lika Osugui, Katia Regina Perez, Ieda Maria Longo-Maugéri, Mario Mariano

**Affiliations:** 1 Discipline of Immunology, Department of Microbiology, Immunology and Parasitology, Universidade Federal de São Paulo, UNIFESP, São Paulo, Brazil; 2 Department of Biophysics, Universidade Federal de São Paulo, UNIFESP, São Paulo, Brazil; 3 Laboratory of Molecular and Cellular Biology, Universidade Paulista, UNIP, São Paulo, Brazil; University of São Paulo, Brazil

## Abstract

The inflammatory response is driven by signals that recruit and elicit immune cells to areas of tissue damage or infection. The concept of a mononuclear phagocyte system postulates that monocytes circulating in the bloodstream are recruited to inflamed tissues where they give rise to macrophages. A recent publication demonstrated that the large increase in the macrophages observed during infection was the result of the multiplication of these cells rather than the recruitment of blood monocytes. We demonstrated previously that B-1 cells undergo differentiation to acquire a mononuclear phagocyte phenotype *in vitro* (B-1CDP), and we propose that B-1 cells could be an alternative origin for peritoneal macrophages. A number of recent studies that describe the phagocytic and microbicidal activity of B-1 cells *in vitro* and *in vivo* support this hypothesis. Based on these findings, we further investigated the differentiation of B-1 cells into phagocytes *in vivo* in response to LPS-induced inflammation. Therefore, we investigated the role of B-1 cells in the composition of the peritoneal macrophage population after LPS stimulation using osteopetrotic mice, BALB/*Xid* mice and the depletion of monocytes/macrophages by clodronate treatment. We show that peritoneal macrophages appear in op/op^(−/−)^ mice after LPS stimulation and exhibit the same Ig gene rearrangement (VH11) that is often found in B-1 cells. These results strongly suggest that op/op^(−/−)^ peritoneal “macrophages” are B-1CDP. Similarly, the LPS-induced increase in the macrophage population was observed even following monocyte/macrophage depletion by clodronate. After monocyte/macrophage depletion by clodronate, LPS-elicited macrophages were observed in BALB/*Xid* mice only following the transfer of B-1 cells. Based on these data, we confirmed that B-1 cell differentiation into phagocytes also occurs *in vivo*. In conclusion, the results strongly suggest that B-1 cell derived phagocytes are a component of the LPS-elicited peritoneal macrophage population.

## Introduction

van Furth and colleagues introduced the concept of a mononuclear phagocyte system (MPS) suggesting that all macrophages, including those appearing in inflammatory foci and residing in tissues under normal steady-state conditions, are derived from monocytes, which differentiate via promonocytes from monoblasts in the bone marrow [1]. Under stimulated conditions, monocytes adhere to activated vascular endothelial cells, migrate into tissues and differentiate into macrophages [2]. The development of monocytic cells and their differentiation into macrophages are supported by macrophage colony-stimulating factor (M-CSF) [3].

Osteopetrotic mice (op/op^(−/−)^ mice) contain a mutation in the coding region of the *cfms* gene, which results in a deficiency of M-CSF [3]. This mutation causes a defect that is associated with osteoclastogenesis and hematopoiesis including a near complete deficiency of monocyte production and a complete deficiency of monocyte-derived macrophages. The daily administration of M-CSF to op/op^(−/−)^ mice increases the number of peripheral blood monocytes, and the differentiation and maturation of monocyte-derived macrophages and osteoclasts is increased to a level found in the normal littermates [4].

Curiously, tissue macrophages develop in various organs and tissues of op/op^(−/−)^ mice [3]. These small, round, and immature cells exhibit an ultrastructure that is characterized by the poor development of intracellular organelles, particularly lysosomal granules. These immature macrophages are present in various organs and tissues of op/op^(−/−)^ mice, particularly in the lungs, spleen and brain. Because op/op^(−/−)^ mice lack functional M-CSF activity and monocytic cells in their peripheral blood, immature macrophages are called “M-CSF-independent macrophages” and are considered to be derived from an earlier macrophage precursor cell than the monocyte [3], [4], [5]. In op/op^(−/−)^ mice, despite the absence of blood monocytes, immature macrophages differentiate from early hematopoietic progenitors without the activity of M-CSF in various organs and tissues [4].

Although various transcription factors are involved in the development and differentiation of hematopoietic stem cells into tissue macrophages, the PU.1 hematopoietic transcription factor is required for the differentiation of early hematopoietic precursors into macrophages and B cells. PU.1-deficient mice die in the fetal stages of development, or they die from septicemia within two days after birth [6], [7]. In these mutant fetuses or neonatal mice, monocyte-derived macrophages are completely absent [6], [7]. Hematopoietic precursors of PU.1-deficient mice did not respond to M-CSF or granulocyte macrophage colony-stimulating factor (GM-CSF) [8]. However, when the mutant mice are rescued by treatment with antibiotics immediately after birth and survive for two weeks, a small number of macrophages develop in various tissues, such as the liver and bone marrow [6]. This result suggests that tissue macrophages develop from early hematopoietic progenitor cells in PU.1- deficient mice and that the development and differentiation of early progenitors into tissue macrophage occurs not only in early ontogeny but also in postnatal life.

Previous studies revealed that pre-B cell lines established in a long-term bone marrow culture differentiate into CD5-positive macrophages *in vitro* [9], [10]. In addition, Takahashi et al. [10] demonstrated that the intravenous treatment of BALB/c and C3H mice with GM-CSF increases the number of CD5-positive macrophages in the peritoneal cavities. These cells were also found in motheaten mutant mice (*me^v^/me^v^*) but were absent in nude, severe combined immunodeficient (*scid*), X-linked immunodeficient (*xid*) and alymphoplasia mice (*aly/aly*) [11]. The *me^v^/me^v^* mice exhibit a tyrosine phosphatase deficiency in their hematopoietic cells, which results in the impairment of T and B cells, but they exhibit an increased number of B-1 cells [11]. After daily intravenous injection with GM-CSF for five days, many CD5^+^ macrophages appeared in the peritoneal cavity and in omental milky spots of normal mice; however, fewer macrophages were detected in op/op^(−/−)^mice [10]. These results indicate that GM-CSF, in combination with M-CSF, induces the development and differentiation of CD5^+^ macrophages in the peritoneal cavity, particularly in the omental milky spots. In the peritoneal cavity of GM-CSF-treated mice, the percentage of hematopoietic progenitor cells doubly positive for CD5 and CD34 or c-kit and macrophage precursor cells doubly positive for CD5 and ER-MP58 or ER-MP20 increased significantly during the development of CD5^+^ macrophages and CD5 B cells, suggesting that CD5^+^ macrophages and B cells may share a bipotential progenitor *in vivo* [10].

Previously, we demonstrated that B-1b cells differentiate to acquire a mononuclear phagocyte phenotype following attachment to a substrate *in vitro* [12]. B-1b cells spontaneously express both myeloid- and lymphoid-restricted transcription factors. When the cells are induced to differentiate into phagocytes, the lymphoid genes encoding E box protein (E2A), early B-cell factor (EBF), and paired box 5 (Pax5) are down-modulated, whereas the expression of genes related to myeloid commitment are sustained. Furthermore, B-1b cell-derived phagocytes (B-1CDPs) decrease immunoglobulin M (IgM) expression but retain the expression of the heavy chain gene variable VH11 or VH12, an immunoglobulin gene rearrangement predominantly expressed by B-1 cells [13]. The maintenance of lymphoid characteristics in B-1CDPs is characteristic of a unique type of phagocyte that is not related to monocyte-derived macrophages. Recent studies have shown that these cells take up apoptotic thymocytes and bacteria *in vitro* and *in vivo* [14], [15]. In addition, Mussalem *et al (in press)* demonstrated that *Propionibacterium acnes* treatment induced the commitment of B-1 cells to the myeloid lineage and their differentiation into phagocytes.

In this study, we demonstrate that “M-CSF-independent macrophages” from op/op^(−/−)^ mice are B-1CDPs. We also demonstrated that B-1CDPs are a source of macrophages after LPS stimulation not only in op/op^(−/−)^ mice but also in normal mice. Additionally, the transfer of B-1 cells to Balb/*Xid* mice confirms that this inflammatory stimulus induces the proliferation of peritoneal resident macrophages, even though these proliferating macrophages are derived from B-1 cells.

## Materials and Methods

### Animals

Breeding pairs of heterozygous B6C3-CSF-1^op/+^ mice were obtained from the Jackson Laboratory (Bar Harbor, ME, USA) and were bred at the animal facilities at Sir William Dunn School of Pathology, University of Oxford. The mutant op/op offspring were clearly identifiable at 10 days of age by the failure of the incisor teeth to erupt and by their domed skull. Toothless mice were separated from their normal siblings at weaning and were fed with powdered mouse food. Homozygous mutant (op/op^−/−^) and heterozygous (op/op^+/?^) littermates were used at 6–8 weeks of age. C57BL6, BALB/c, and BALB/*Xid* male mice (6–8 weeks old) were bred locally at Universidade Federal de São Paulo under standard pathogen-free conditions.

### Ethics Statement

All experiments were conducted with the approval of the institutional animal care and use committee. The Comitê de Ética em Pesquisa (CEP) da Universidade Federal de São Paulo/Hospital São Paulo (ID number: CEP 1015/2005) approved this study.

### *In vivo* LPS injection

Mice were inoculated intraperitoneally (i.p.) with 10 µg of lipopolysaccharide (LPS - *Escherichia coli* serotype O26:B6, 90–99% pure; Sigma–Aldrich, St. Louis, MO, USA) in 200 µL of phosphate buffered saline (PBS) solution or with PBS alone and were killed 3 days later for flow cytometric analysis of the peritoneal macrophages. In some experiments, carboxyfluorescein succinimidyl ester (CFSE) was injected simultaneously with LPS and control PBS for the analysis of monocyte migration or *in vivo* proliferation.

### Peritoneal cells

Peritoneal cells were collected from the abdominal cavity of mice by repeated lavage with 2 ml of RPMI-1640 medium (Sigma, St Louis, MO). Cell viability was evaluated using the trypan blue dye exclusion method. The absolute cell numbers were determined, and the cells were submitted to flow cytometry analysis, cell purification and DNA extraction.

### Flow Cytometry Analysis

The peritoneal cells were stained with monoclonal antibodies and were analyzed as described below. The cell suspensions were pre-incubated with anti–CD16/CD32 mAb to block FcγRII/III receptors and stained on ice for 15 minutes with the following fluorochrome conjugated mAbs: fluorescein isothiocyanate (FITC)- or peridinin clorophyll cyanine dye 5 (PerC-Cy5)-labeled anti-mouse CD19, phycoerythrin (PE)-labeled anti-mouse CD23, peridinin clorophyll protein (PerCP)-labeled anti-mouse CD5, allophycocyanin (APC) or Pacific blue-labeled anti-mouse CD11b, PE- or FITC-labeled IgM and Pacific orange-labeled F4/80. Antibodies were purchased from BD Bioscience, with the exception of the Pacific blue-labeled anti-mouse CD11b and the Pacific orange-labeled F4/80, which were from Invitrogen. Cell staining was performed in accordance with the manufacturer's protocol, and the combinations of different antibodies used are indicated in each figure. Flow cytometric detection was performed on FACS Canto, FACS Calibur (Becton Dickinson) or Attune® Acoustic Focusing Flow Cytometer (Life Technology, Applied Biosystems) instruments, and analysis was performed using Flow Jo software (Tree Star). Fluorescence-minus-one (FMO) controls were used to distinguish positively stained from autofluorescent cells.

### Peritoneal Macrophage Purification

Non-B cells were separated from peritoneal cells using the MiniMACS magnetic bead system (Miltenyi Biotech, Bergisch Gladbach, Germany). Approximately 10^7^ peritoneal cells were incubated with monoclonal FITC anti-mouse CD19 and FITC anti-IgM (Pharmingen, San Diego, CA) diluted in a saline solution containing 5% bovine serum albumin (BSA) and 2 mM ethylenediaminetetra-acetic acid (EDTA). After washing with the same dilution buffer, anti-rat immunoglobulin G (IgG) coupled to iron (Fe) (Miltenyi Biotec) was added to the cell suspension for 45 min at 4°C. The cells were passed through a 25 MS positive selection column (Miltenyi Biotec) to capture the B cells. Non-B cells were collected and after cell separation; the cells were stained on ice for 15 minutes with APC-labeled anti-mouse CD11b, PE-streptavidin and biotin anti-F4/80 and were submitted to FACS analysis. Non-B cell exclusion was confirmed by flow cytometry analysis and after confirming the absence of CD19^+^IgM^+^ cells, the non-B cells were submitted to Ig gene rearrangement experiments.

### B-1 cell sorting and adoptive transfer to BALB/Xid mice

Peritoneal B-1 cells were purified from peritoneal lavages pooled from 10 mice, and the cells were sorted using a FACSAria III Cell Sorter (BD Biosciences) using the parameters described in Figure S1. Briefly, doublets were excluded from total peritoneal cells by the analysis of FSC-A×FSC-H parameters. Only single cells were considered to delimit the lymphocyte gate. CD19^+^CD23^−^ cells (B-1 cells) were sorted according to the expression of CD19 and CD23 on lymphocytes. After cell-sorting, purified B-1 cells were stained with CFSE before transfer to BALB/*Xid* mice. Next, 1×10^6^ cells were transferred to the peritoneal cavity of BALB/*Xid* mice simultaneously with the injection of LPS. After 3 days, the peritoneal cells were collected for analysis. In some experiments, clodronate treatment was performed before the transfer of the B-1 cells.

### Ig gene rearrangement analysis

Non-B cells obtained as described above were submitted to DNA extraction. DNA amplification was performed using a Superscript Cells Direct cDNA Synthesis System (Invitrogen). The PCR products were selected for VH11 and VH12 as described by Popi et al. [13]. The cDNAs were selected based on the expression of housekeeping genes. B cells were used as the positive control.

### CFSE staining

The B-1 cells were stained with CFSE prior to their transfer. Briefly, purified B-1 cells (1×10^6^ cells/ml) were incubated with 50 µM CFSE (Molecular Probes) in prewarmed PBS for 5 min at 37°C and washed twice with RPMI medium supplemented with 10% fetal bovine serum. In other experiments, CFSE (25 µM) was injected directly into the peritoneal cavity of mice to stain the resident peritoneal cells. To confirm that only peritoneal cells were stained by CFSE, after 1 hour, the blood and spleen cells were analyzed, and no staining was observed in these samples.

### Liposome preparation

Clodronate was entrapped in liposomes by ether injection as described by Lepique et al [16]. Depletion of macrophages was performed before the LPS stimulation. The mice were i.p injected twice with 200 µL of the liposome preparation (12 µg of clodronate) at 72 and 24 h before LPS injection. In some experiments, the B-1 cell transfer was performed simultaneously with the injection of LPS.

### Statistical analysis

All data shown are the means (SEM). Significant differences between sample means were determined using the Student's *t* test or two-way ANOVA.

## Results

### Macrophages and B-1 cells are reduced in peritoneal cavity of op/op^(−/−)^ mice

**Figure 1:**
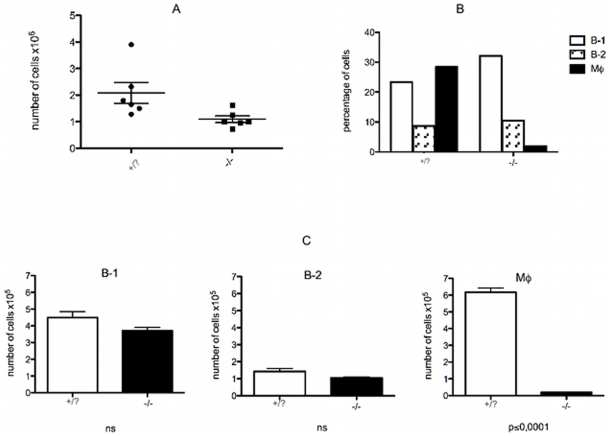
Characterization of peritoneal cell subpopulations in the peritoneal cavity of op/op^(−/−)^ mice. (A) The number of cells in the peritoneal cavity of op/op^(−/−)^ mice is significantly reduced when compared to the littermates. Peritoneal cells were collected from op/op^(−/−)^ mice and their normal littermates (op/op^(+/?)^), and the cells were counted (p<0.01). (B) Percentage of B-1 cells, B-2 cells and macrophages in the peritoneal cavity of op/op^(−/−)^ mice and littermates (op/op^(+/?)^). (C) Absolute number of B-1 cells (ns), B-2 cells (ns) and macrophages (Mϕ - p<0.0001). Cell populations were determined by flow analysis based on the following surface marker expression: B-1 cells (CD23^−^CD19^+^CD11b^+^), Mϕ (CD11b^+^F4/80^+^CD19^−^), and B-2 (CD23^+^CD19^+^CD11b^−^). Graphs are representative of two independent experiments using 6 mice per group per experiment.

It has been reported that the number of hematopoietic cells in op/op^(−/−)^ mice is lower than in op/op^(+/?)^ or wild type mice [17], [18]. Cells were collected from the peritoneal cavity of both op/op^(−/−)^ mice and their normal littermates (op/op^(+/?)^). As shown in Figure 1A, the total number of peritoneal cells in the op/op^(−/−)^ mice was lower when compared with op/op^(+/?)^ mice. The cell populations from the peritoneal cavity of op/op^(−/−)^ and op/op^(+/?)^ mice were analyzed based on surface marker expression. Quantitative and qualitative analysis was performed to characterize B cells (IgM^low^CD23^+^CD19^+^CD11b^−^CD5^−^), macrophages (IgM^−^CD11b^+^F4/80^+^CD19^−^CD5^−^) and B-1 cells (IgM^high^CD23^−^CD19^+^CD11b^+^CD5^+/−^). Confirming data previously described in the literature [17], the number of macrophages in the op/op^(−/−)^ mice was drastically reduced (Figure 1B and C). Analysis of the B cell subsets revealed a small reduction in the B-1 cell population (not significant), and no differences in B cell numbers were detected when op/op^(−/−)^ mice and their littermates were compared (Figure 1B and C).

### LPS increases the number of B-1 cells and macrophages in the peritoneal cavity of op/op^(−/−)^ mice

**Figure 2:**
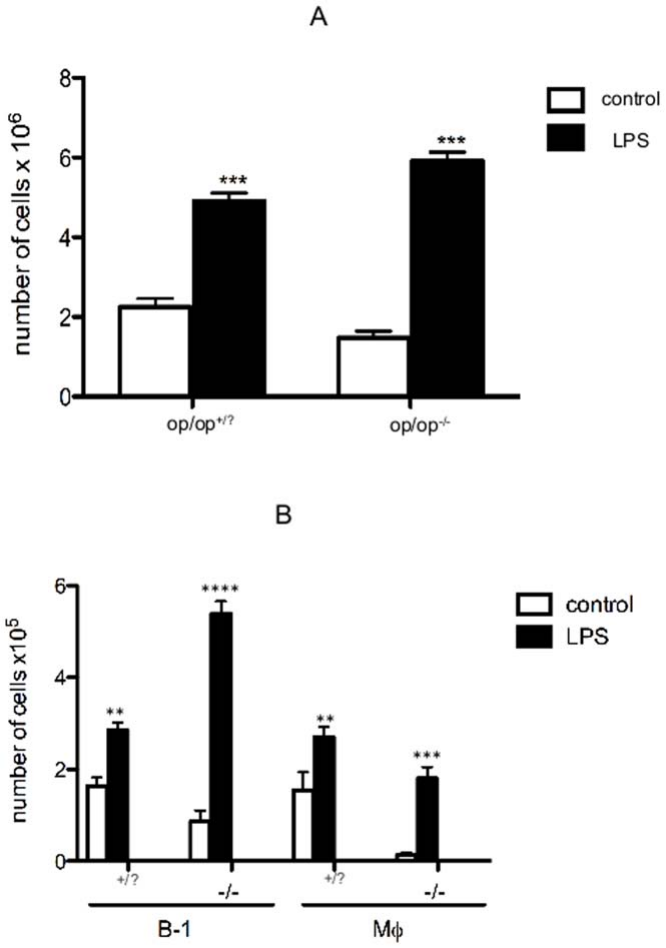
LPS increases the number of B-1 cells and macrophages in the peritoneal cavity of op/op^(−/−)^ mice. (A) Number of peritoneal cells collected and counted individually from 6 mice (op/op^(−/−)^) and their littermates (op/op^(+/?)^) in control or LPS-treated groups. (B) Absolute number of B-1 cells and macrophages in untreated control (□) or LPS stimulated (▪) littermates op/op^(+/?)^ and op/op^(−/−)^ mice. Cell populations were determined by flow analysis based on surface marker expression: B-1 cells (CD23^−^CD19^+^CD11b^+^) and Mϕ (CD11b^+^F4/80^+^CD19^−^). Flow cytometric analysis showed significantly increased percentages of B-1 cells in both mice lineages after LPS stimulation when compared to untreated mice. LPS also increased the number of macrophage cells in both groups of mice. Significant differences between the sample means are indicated as follows: **p<0.05, ***p<0.01, and ****p<0.001. The results are representative of two independent experiments.

M-CSF-derived macrophages from bone marrow progenitors generate large amounts of TNF-α after stimulation with LPS [19]. After LPS stimulation, the phagocytic rate in peritoneal macrophages isolated from the op/op^(−/−)^ mice was significantly reduced compared with the macrophages isolated from the wild type mice [20]. These observations support the hypothesis that the functional heterogeneity of peritoneal macrophages exists between op/op^+/+^ mice and op/op^−/−^ mice [20]. Next, we investigated B-1 cells (IgM^high^CD23^−^CD19^+^CD11b^+^CD5^+/−^) and macrophages (IgM^−^CD11b^+^F4/80^+^CD19^−^CD5^−^) in the peritoneal cavity of op/op^(−/−)^ mice after LPS stimulus *in vivo*. Following LPS stimulation, a significant increase in the total number of peritoneal cells was observed (Figure 2A). Analysis of the different peritoneal cell subtypes revealed a larger increase in the B-1 cell population from op/op^(−/−)^ mice after LPS treatment compared with the increase observed in op/op^(+/?)^ mice. As expected, the number of macrophages in the op/op^(+/?)^ mice increased following the inflammatory stimulus. Intriguingly, the LPS stimulus also induced an increase in the “macrophage” population in op/op^(−/−)^ mice, even though these animals have a severe deficiency of monocyte-derived macrophages. It is important to emphasize that in this study, the macrophages were characterized based on surface marker expression, which means that these cells were CD11b and F4/80^(low or high)^ double positive cells, concomitant with the absence of CD19, IgM and CD5. Based on the upsurge in the macrophage population in the op/op^(−/−)^ mice, we questioned the origin of these phagocytes. We have demonstrated previously that B-1 cells differentiate into phagocytes (B-1CDP) *in vitro* [12], [13]. After differentiation, B-1CDPs lose their B cell marker expression and express monocyte-derived macrophages surface markers. Therefore, we hypothesized that the inflammatory stimulus induced B-1 cell differentiation into phagocytes *in vivo* and that the enlargement of the macrophage population may be related to the emergence of B-1CDPs.

### “Macrophages” from op/op^(−/−)^ mice contain an Ig gene rearrangement

**Figure 3:**
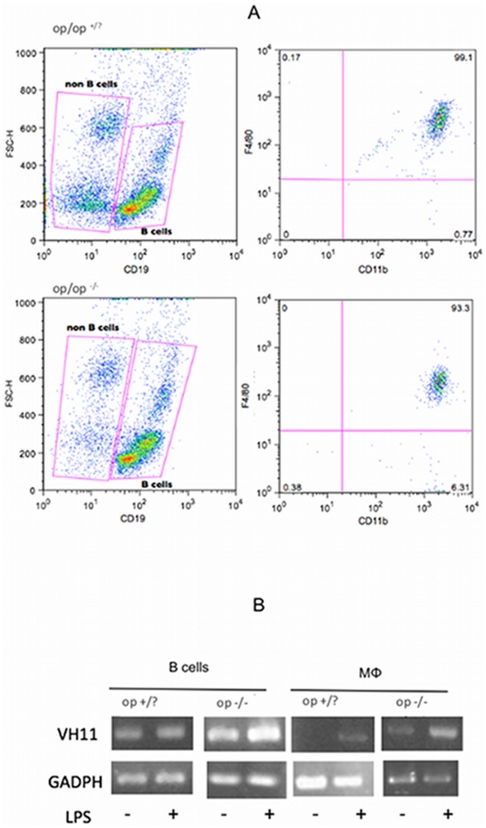
Elicited peritoneal “macrophages” from op/op^(−/−)^ mice exhibit Ig gene rearrangements. A) Flow cytometry analysis of total peritoneal cells to distinguish B cells (CD19^+^) and non-B cells (CD19^−^). After cell purification, the CD19^+^ cells and the CD19^−^ cells were collected separately. The right dot plots show that non-B cells are F4/80^+^CD11b^+^ and are thus characterized as macrophages. B) Analysis of heavy chain variable gene VH11 expression by peritoneal B cells and macrophages from unstimulated or LPS-stimulated op/op^(+/?)^ and op/op^(−/−)^ mice. The PCR products were visualized in agarose gels by ethidium bromide staining. The amount of cDNA input was normalized by analyzing the control GADPH transcripts. The results are representative of three independent experiments using 8 mice per group per experiment.

Considering that in op/op^(−/−)^ mice monocyte-derived macrophages are virtually absent, and LPS increased the number of cells characterized as “macrophages”, we investigated if these cells were B-1 cell-derived phagocytes. Because B-1CDPs contain the VH11 Ig gene rearrangement [13], we investigated the probable expression of this Ig gene rearrangement in macrophages from the peritoneal cavity of op/op^(−/−)^ mice. Peritoneal cells were collected from the peritoneal cavity of mice following administration of the LPS stimulus, and B cells (CD19^+^IgM^+^) were separated from the non-B cells (CD19^−^IgM^−^) using microbeads (Figure 3A). The non-B cells were collected and were submitted to cytometric analysis. The results showed that the peritoneal non-B cells were IgM^−^CD19^−^CD11b^+^F4/80^+^ (99,98%), and they were characterized as “macrophages” (Figure 3A). Using methods described in the literature [13], the Ig gene rearrangement in “macrophages” and B cells was analyzed. The VH11 Ig gene rearrangement was not found in macrophages from op/op^(+/?)^ mice, which supports the current concept that the majority of these cells result from monocyte differentiation. However, analysis of the Ig gene rearrangement in “macrophages” from op/op^(−/−)^ mice clearly demonstrates that these cells contain the VH11 Ig gene rearrangement that is found in B-1 cells (Figure 3B). These results strongly suggest that B-1 cell-derived phagocytes (B-1CDPs) are a component of “MCSF independent macrophages” in the peritoneal cavity after LPS stimulation.

### The increase in macrophages following LPS stimulation occurs independently of monocytes

Although these data reinforce the hypothesis that B-1 cells differentiate into phagocytes *in vivo*, there is a possibility that the large increase in macrophages observed after the LPS stimulus results from the expansion of the peritoneal resident population rather than from monocyte recruitment.

**Figure 4:**
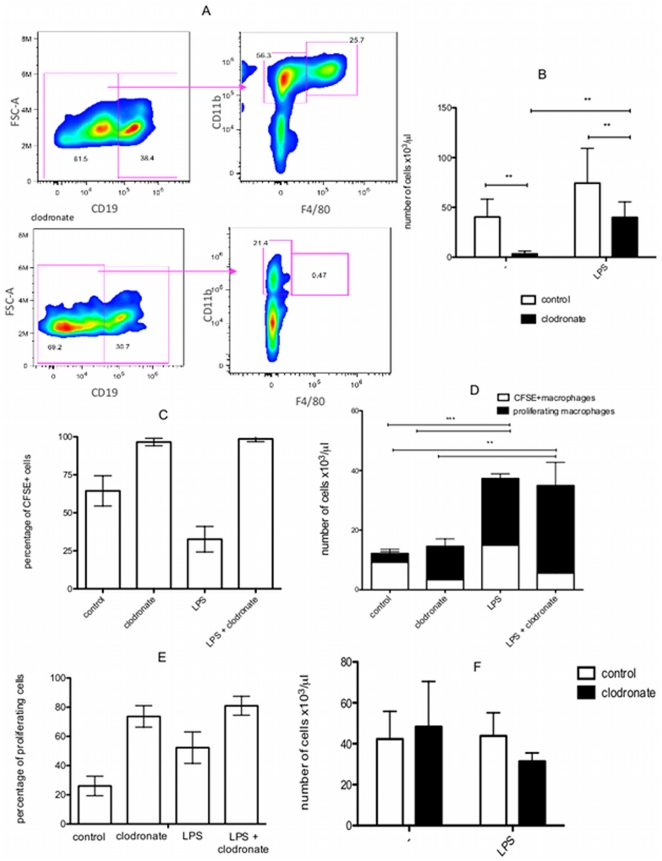
Clodronate treatment did not impede the proliferation of resident peritoneal “macrophages” from BALB/c mice treated with LPS. A) Peritoneal cells were collected from untreated and clodronate-treated mice 3 days after the last clodronate injection. Non-B cells were selected based on the total peritoneal CD19^−^ cells, and the CD11b^+^F4/80^+^ cells were considered macrophages. This CD11b^+^F4/80^+^ cell population was not observed after clodronate treatment. B) Absolute number of peritoneal macrophages (CD19^−^CD11b^+^F4/80^+^) from non-clodronate treated (control group □) or clodronate-treated (▪) BALB/c mice after LPS or saline (−) injection. C) Percentage of CFSE^+^ cells present in the peritoneal macrophage population (CD19^−^CD11b^+^F4/80^+^) in BALB/c mice subjected to different treatments: control (without clodronate+saline injection); clodronate (clodronate+saline); LPS (without clodronate+LPS injection) and LPS+clodronate (clodronate+LPS). The same group legend was used in figure 4D and 4E. D) Absolute number of CFSE^+^ macrophages (CFSE^+^CD19^−^CD11b^+^F4/80^+^) showing high CFSE fluorescence (non-proliferating cells; CFSE^+^macrophages) and the decay in CFSE fluorescence (proliferating macrophages). E) Percentage of proliferating macrophages (CFSE^dim^) detected in different treated BALB/c mice. F) Absolute number of peritoneal B-1 cells (CD23^−^CD19^+^CD11b^+^) in non-clodronate treated (control group □) or clodronate-treated (▪) BALB/c mice after LPS or saline (−) injection. **p<0.01 and ***p<0.001 when the indicated groups are compared. The results are representative of two or three independent experiments using 5 mice per group per experiment.

To determine the role of monocytes in macrophage accumulation, we injected clodronate-loaded liposomes intraperitoneally (i.p.). Clodronate treatment depletes monocytes and blocks tissue infiltration by macrophages in a variety of inflammatory settings [21], [22], [23]. First, we confirmed that the clodronate treatment protocol adopted in this study resulted in an effective and sustained depletion of peritoneal macrophages. As expected, clodronate-treated mice remained depleted of peritoneal macrophages for at least 5 days after the last clodronate-liposomes injection (Figure 4A). Considering this depletion, 24 hours after the last clodronate-liposomes injection, BALB/c mice were injected with LPS. To discriminate resident peritoneal cells from newly migrating cells, CFSE was injected into the peritoneal cavity prior to LPS injection. Thus, the resident cells were now CFSE^+^ cells. After 3 days, the peritoneal macrophage population was analyzed. First, peritoneal B cells were excluded based on their CD19 expression. Subsequently, macrophages were identified by their expression of CD11b and F4/80. As expected, this cell population increased after LPS stimulation in the non-clodronate treated mice (control group). Interestingly, the LPS stimulus induced an increase in the macrophage population, even though monocyte/macrophage depletion was induced by clodronate (Figure 4B). These data corroborate the increase in the macrophage population after LPS stimulation in op/op^(−/−)^ mice.

We also analyzed the percentage of macrophages (CD11b^+^F4/80^+^ cells) staining with CFSE, which were considered resident macrophages (Figure 4C). We observed that 96% of the macrophages were CFSE^+^ in the clodronate-treated mice compared to 64.4% in the non-treated mice (control group). This data confirms that clodronate treatment causes macrophage depletion and the inhibition of monocyte migration to the peritoneal cavity. Furthermore, after stimulation with LPS, only 32% of the peritoneal macrophages were CFSE^+^, whereas in the clodronate treated group, this percentage increased to 98%. These data confirm that monocyte-derived macrophages were eliminated by clodronate treatment (Figure 4C). Furthermore, compared with the control group, we observed a smaller percentage of CFSE^+^ macrophages in the peritoneal cavity of LPS-stimulated mice indicating that the LPS stimulus induced monocyte migration and differentiation. However, when the monocyte-macrophage population was depleted, an increase in the number of peritoneal resident cells was observed following LPS stimulation. Based on this observation, we concluded that the enlargement of the macrophage population after LPS stimulation occurs not only as consequence of monocyte migration but also as result of an increase in one of the resident cell populations in the peritoneal cavity.

Given that CD11b^+^F4/80^+^CFSE^+^ cells are resident peritoneal macrophages, macrophage proliferation was determined based on CFSE decay. Therefore, CD11b^+^F4/80^+^CFSE^+^ cells were divided into non-proliferating cells (CFSE^high^, CFSE^+^ macrophages) and proliferating macrophages (CFSE^dim^). Undoubtedly, the LPS stimulus induced the proliferation of resident macrophages; 52% of the resident macrophages exhibited diminished CFSE fluorescence in the LPS-treated group. More importantly, after peritoneal macrophage depletion by clodronate treatment, a pronounced proliferation of CD11b^+^F4/80^+^ cells was still observed (80% of resident macrophages were proliferating in the clodronate+LPS-treated group) (Figure 4D and E). These data reinforce the theory that the LPS stimulus provoked an increase in the macrophage population, at least partially, by inducing the proliferation of a peritoneal resident cell subtype. Furthermore, this increase was observed despite the depletion of the monocyte-derived macrophage population. It is reasonable to assume that other peritoneal resident cells are induced by LPS to differentiate into “macrophages” and are responsible for the increase in this population. These data corroborate our hypothesis that LPS stimulates the differentiation of B-1 cells into phagocytes *in vivo*. It is important to note that B-1 cells are not affected by clodronate treatment (Figure 4F).

### B-1CDPs are part of the LPS-elicited peritoneal macrophage population

**Figure 5:**
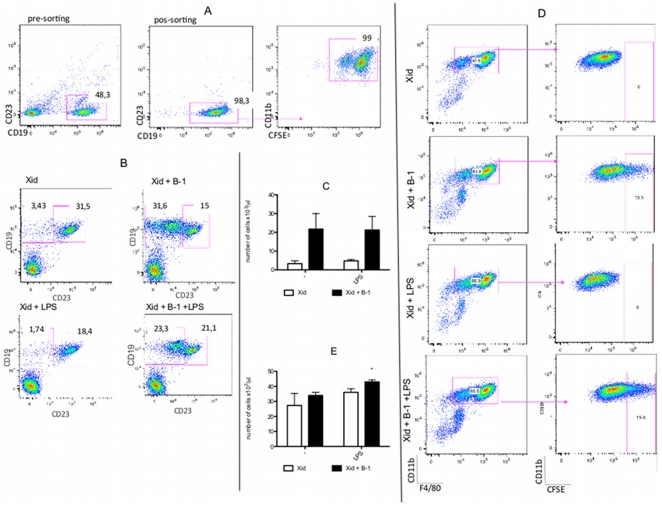
LPS induces B-1CDP differentiation and proliferation. A) Analysis of B-1 cell purification and CFSE^+^ staining. B-1 cells were obtained from the total peritoneal cells based on the selection of CD19^+^CD23^−^ cells as described in Figure S1. The first dot plot represents the B-1 cell gate selected to perform cell sorting. The second dot plot illustrates B-1 cells after cell sorting. Subsequently, these cells were subjected to CFSE staining as demonstrated in the last dot plot. B) Analysis of expression of CD19 and CD23 by peritoneal lymphocytes confirms the transfer of B-1 cells to BALB/*Xid* mice. B-1 cells are almost absent in BALB/*Xid* and BALB/*Xid*+LPS mice but are present in significant numbers in BALB/*Xid*+B-1 and BALB/*Xid*+B-1+LPS mice, indicating the efficiency of the B-1 cell transfer. C) Absolute number of B-1 cells (CD19^+^CD23^−^CD11b^+^) in the peritoneal cavity of BALB/*Xid* (□) and BALB/*Xid*+B-1 (▪) after LPS or saline (−) injection. p<0.001 when the LPS-stimulated group is compared to the non-stimulated group. D) Analysis of the presence of B-1CDP (CFSE^+^ cells) in the peritoneal macrophage population (CD19^−^CD11b^+^F4/80^+^) from BALB/*Xid*, BALB/*Xid*+B-1, BALB/*Xid*+LPS and BALB/*Xid*+B-1+LPS mice. E) Absolute number of peritoneal macrophages (CD19^−^CD11b^+^F4/80^+^) from BALB/*Xid* mice (□) and BALB/*Xid*+B-1 mice (▪) after LPS or saline (−) injection. * p<0.01 when the indicated group is compared to all groups. The results are representative of two independent experiments using 5 mice per group per experiment.

To confirm the hypothesis that after LPS stimulation, the macrophage population is composed of B-1 cell-derived phagocytes, the peritoneal cell population of BALB/*Xid* mice was analyzed. To evaluate the participation of B-1 cells in the composition of the macrophage population, B-1 cells were purified, stained with CFSE and transferred to the peritoneal cavity of BALB/*Xid* mice prior to administration of the LPS stimulus. Figure 5A illustrate the acquisition of purified B-1 cells, corresponding to 98,3% CD19^+^CD23^−^ cells sorted. After CFSE staining, B-1 cells (CD19^+^CD23^−^CFSE^+^ cells) were transferred intraperitoneally (i.p.) to BALB/*Xid* mice simultaneously with the LPS injection. After 3 days, the peritoneal cells from these mice were analyzed. Only 3.43±1.23% of the lymphocytes present in the peritoneal cavity of BALB/*Xid* mice were B-1 cells, which was equivalent to 0.27% of the total number of cells. After transfer of the B-1 cells, this percentage increased to 31.6±2.8% of the peritoneal lymphocytes (5.12% of the total cells) (Figure 5B). The increase in the B-1 cell population after cell transfer to BALB/Xid mice is demonstrated in Figure 5C.

The macrophage population was also analyzed after B-1 cell transfer to BALB/*Xid* mice. Using this strategy, we analyzed the participation of B-1 cell-derived phagocytes in the composition of the macrophage population. The CD11b^+^F4/80^+^ cells were selected, and the number of CFSE^+^ cells (B-1 cells) in this population was determined (Figure 5D). After transfer of the B-1 CFSE^+^ cells to BALB/*Xid* mice, we determined that at least 15% of the macrophages were derived from B-1 cells (CFSE^+^). No difference was observed in the absolute number of macrophages when BALB/*Xid* and BALB/*Xid*+B-1 were compared. However, after LPS stimulation, a subtle increase (16.66%) in the macrophage population of BALB/*Xid*+B-1 mice was observed when compared to non-stimulated BALB/*Xid* mice (Figure 5E). The data shown here confirm that B-1 cells differentiate into phagocytes *in vivo*, and support the hypothesis that B-1CDP is a component of the macrophage population after LPS stimulation in the peritoneum.

**Figure 6:**
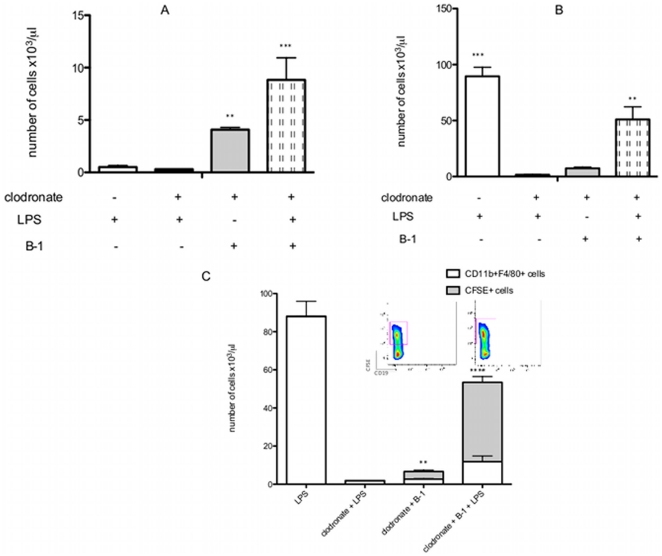
B-1CDPs are one of the components of the peritoneal macrophage population after LPS stimulation. A) Absolute number of peritoneal B-1 cells (CD19^+^CD23^−^CD11b^+^) detected in BALB/*Xid* mice submitted to the specified treatment. ** p<0.01 and ***p<0.001 when the indicated groups are compared with the non-marked groups. p<0.001 when the two last groups are compared. B) Absolute number of peritoneal macrophages (CD19^−^CD11b^+^F4/80^+^) detected in BALB/*Xid* mice submitted to the specified treatment. ** p<0.01 and ***p<0.001 when the indicated groups are compared with the non-marked groups. C) Absolute number of macrophages (CD19^−^CD11b^+^F4/80^+^) and the number of B-1CDPs (CFSE^+^ cells) detected in this population from BALB/*Xid* mice submitted to the specified treatment. **p<0.01 and ****p<0.001 when the CFSE^+^ cell number of the indicated groups are compared with all other groups. The results are representative of two independent experiments using 4 or 5 mice per group per experiment.

To demonstrate definitively that B-1CDPs contribute to increasing peritoneal macrophages after LPS stimulation, BALB/*Xid* mice were treated with clodronate as described above. At 24 hours following the last clodronate injection, purified B-1 CFSE^+^ cells were transferred to the peritoneal cavity and the LPS was injected. As shown in Figure 6A, the B-1 cell population was present in BALB/*Xid* mice after transfer. Once again, it is important to note that clodronate treatment did not affect the B-1 cells, despite the efficiency of clodronate treatment, which was confirmed by complete deletion of the macrophage population in BALB/*Xid* mice (Figure 6B). Analyzing the macrophage population after LPS stimulation, we observed a prominent CD11b^+^F4/80^+^ cell population in the peritoneal cavity of BALB/*Xid* mice, whereas these cells were absent when mice were treated with clodronate. In contrast, CD11b^+^F4/80^+^ cells were still observed in the peritoneal cavity of BALB/*Xid* mice that received B-1 cells (CFSE^+^) by adoptive transference, even after clodronate treatment. Quantitative analysis of CFSE^+^ cells in the CD11b^+^F4/80^+^ cell population confirms that B-1 cell-derived phagocytes are an important component of the macrophage population after LPS stimulation (Figure 6C). After LPS stimulation, the majority of macrophages found in the peritoneum of BALB/*Xid* after clodronate treatment was CFSE^+^ (81,74%); hence, they were derived from B-1 cells. The insert in Figure 6C illustrates the presence of CFSE^+^ cells that constitute the macrophage population. In agreement with published data [13], CD19 was not expressed by the B-1 cells after their differentiation into phagocytes. These data emphasize the differentiation of a B-1 cell into a phagocyte *in vivo* after LPS stimulation.

## Discussion

The concept of the mononuclear phagocyte system (MPS) proposed by van Furth et al. [1] has been constantly revisited. According to the MPS concept, all macrophages are derived from monocytes. However, according to phylogenetic and ontogenetic points of view, macrophages emerge before the development of monocytic cells. With regard to ontogeny, primitive and fetal macrophages first develop in the yolk sac where monocytes are not detected, and this indicates that in early hematopoiesis, macrophages arise bypassing monocyte development [24], [25]. In addition, hematopoietic stem cells have been shown to differentiate without passing through the developmental stages of monocytic cells. Data here described question the MPS concept, pointing out that the development and differentiation of macrophages occurs not in a single cell lineage, but possibly through multiple pathways.

The differentiation pathway of macrophages via monocytes is supported by M-CSF and inflammatory signals. It has been demonstrated that in op/op^(−/−)^ mice, this differentiation pathway is completely blocked. However, results show that this blockage does not occur in a number of primitive macrophages or in immature tissue macrophages suggesting the heterogeneity of macrophage origin [3], [24].

Reports in the literature support the presence of M-CSF-independent macrophages in op/op^(−/−)^ mice that have differentiated not from monocytes but from early (unidentified) progenitors [3], [4], [5]. M-CSF-independent macrophages are considered immature macrophages, and it is thought that they are present in various tissues in the absence of inflammatory signals or that they rapidly migrate in response to an inflammatory stimulus. Furthermore, M-CSF-independent macrophages are described as small round cells characterized by an ultrastructure containing a low intracellular organelle content, particularly lysosomal granules [3]. It is interesting to note the similarities between the morphology of M-CSF-independent macrophages and that described for B-1 cells [26]. Based on the data reported in this study, we speculate that the M-CSF-independent macrophages described in the literature could be derived from B-1 cells.

In this study, we demonstrate that the deficiency of the *cfms* gene in op/op^(−/−)^ mice does not influence the peritoneal B-1 cell population, in the same way as it affects macrophage population. Furthermore, when compared with normal littermates, B-1 cell proliferation levels increase with LPS stimulation in op/op^(−/−)^ mice. Intriguingly, intraperitoneal LPS stimulation resulted in a significant enlargement in the macrophage population in op/op^(−/−)^ mice. As discussed above, it is reasonable to suggest two origins for macrophages, those derived from monocytes and those “macrophages” derived from B-1 cells, which are the predominant macrophages found in op/op^(−/−)^ mice. This hypothesis is supported by the Ig gene rearrangement (VH11) found in “macrophages” from op/op^(−/−)^ mice and their normal littermates. Consistent with our previous data, B-1 cell-derived phagocytes contain the VH11 or VH12 Ig gene rearrangement, which is typical of B-1 cells [13]. Importantly, the levels of VH11 detected in “macrophages” from op/op^(−/−)^ mice after LPS stimulation is higher in comparison with the op/op^(+/+)^ levels, indicating that “macrophages” from op/op^(−/−)^ mice are predominantly formed by B-1 cell-derived phagocytes. The transfer of B-1 CFSE^+^ cells to the peritoneal cavity of BALB/*Xid* mice and the presence of CD11b^+^F4/80^+^CFSE^+^ macrophages support this hypothesis.

Our data also confirm that following an inflammatory stimulus, the macrophage population in the peritoneal cavity increases not only due to monocyte migration but also because of proliferation of resident peritoneal cells. As indicated by Jenkins *et al.* [27], pleural macrophages proliferate *in situ* in response to Th2 inflammatory signals. The study proposes that macrophage proliferation *in situ* is an alternative mechanism of inflammation that increases the phagocyte population for the performance of critical functions, such as parasite sequestration or wound repair, in the absence of potentially damaging cell recruitment. However, we demonstrated that increase in macrophage population after LPS stimulation is a consequence of a proliferation in one of the resident peritoneal cells, other than monocytes. Moreover, the transfer of B-1 CFSE^+^ cells to BALB/*Xid* mice clearly demonstrated that LPS treatment leads to B-1 cell proliferation and differentiation into phagocytes. Based on this data, we question if monocyte-derived macrophages proliferate *in vivo*. Furthermore, we reinforced our hypothesis by considering more than one origin for peritoneal macrophages. The definitive role of B-1CDPs in the inflammatory response and their function as macrophages is under investigation.

The discovery that B-1b cells can be obtained from adherent mouse peritoneal cell cultures and that these cells spontaneously differentiate into mononuclear phagocytes that are unrelated to monocytes introduced a new candidate for the mononuclear phagocytic system [12], [13]. It is important to note that B-1b cells migrate from the peritoneal cavity to non-specific inflammatory lesion sites just as monocytes migrate from the bone marrow. Recent studies in our laboratory support the existence of B-1 cell-derived phagocytes *in vivo* [15]. These cells ingest apoptotic bodies and *E. coli* bacteria *in vivo*, although less effectively than macrophages. Other authors also demonstrated the phagocytic and microbicidal ability of peritoneal B-1 cells [28]. Consistent with these data, Borrello and Phipps [29], [30] demonstrated that when splenic B-1a cells were co-cultivated with fibroblasts, they became phagocytes, which they termed B/macrophages [31]. These authors proposed that in addition to the monocytic origin of macrophages as defined by the concept of the mononuclear phagocytic system, macrophages could also originate from B-1 cells.

It has been demonstrated that the pre-B cell line J13 differentiates into CD5-positive “macrophages” after culturing for 1 month in the presence of GM-CSF [9], [10]. Furthermore, the authors analyzed different mouse strains, including *me^v^/me^v^*, *nude*, *scid* and *xid* mice, to detect CD5-positive “macrophages” *in vivo*. CD5-positive macrophages were observed only in the *me^v^/me^v^* mice. Importantly, the *me^v^/me^v^* mice are deficient in B and T cells but have B-1 cells in abundance, which is in contrast to other B-1 cell-deficient strains. In this study, we observed that clodronate treatment abolished macrophage cells in the peritoneal cavity of BALB/c and BALB/*Xid* mice, even after LPS stimulation. The observation that the “macrophage” population was present in the peritoneal cavity after clodronate treatment only when B-1 cells were transferred to BALB/*Xid* mice supports the hypothesis that LPS-elicited macrophages originate not only from monocyte-derived macrophages but also from B-1 cell derived phagocytes. Additionally, experiments using fetal liver or bone marrow transplants in *scid* mice have confirmed that CD5-positive “macrophages” develop from B-1 cells and are detected in milky spots and the peritoneal cavity [32].

Plytycz and Seljelid [33] proposed that B-1 cells are ‘living fossils’, and suggested they are primordial cells in the phylogenesis of the hematopoietic system. Extensive adoptive transfer studies [34], [35], [36], [37] suggest that the progenitors of B-1a cells reside in the fetal omentum and fetal liver and are missing in the bone marrow of adult mice. Furthermore, B-1 cells appear in the para-aortic splanchnopleura of 9.5 (dpc) embryo mice. A “primitive” macrophage population that is derived from the yolk sac and is not related to monocytes has been detected in mouse embryos (7 dpc). Monocytes appear in the embryonic liver on day 11 (pc) when definitive hematopoiesis occurs [24], [38]. Coincidently, B-1 cells appear in mammals at a similar stage of development, before the hematopoietic system develops.

The biphenotypic B/macrophage lineage precursor present in mammals suggests a close evolutionary relationship between B cells and monocyte-derived macrophages, implying a possible common phylogenetic predecessor with attributes of both cell types. Reinforcing this hypothesis is the demonstration by Li and colleagues [39] that lymphocytes in teleostean fish, a primitive animal, are biphenotypic.

The data presented here demonstrate a relationship between B-1CDPs and M-CSF-independent macrophages in op/op^(−/−)^ mice. The hypothesis that B-1 cell-derived phagocytes are related to the M-CSF-independent macrophage population is also supported by the absence of the M-CSF receptor in B-1 cells [13]. Despite the fact that B-1CDPs acquire MCSF-R after differentiation *in vitro*, the absence of the receptor in B-1 cells suggests that M-CSF do not act as an important factor to induce differentiation of B-1 cells into phagocytes. However, the role of M-CSF signaling in B-1 cell differentiation into phagocytes needs further investigation.

Furthermore, depletion of macrophages by clodronate treatment in a normal mouse strain (BALB/c) confirms the participation of B-1 cell-derived phagocytes as a component of the peritoneal macrophage population. Moreover, detection of CFSE^+^ macrophages in the peritoneal cavity of BALB/*Xid* mice after the transfer of B-1 CFSE^+^ cells reinforces the hypothesis that B-1 cells differentiate into phagocytes *in vivo* after LPS stimulation. The current knowledge about B-1 cells and their ability to differentiate into phagocyte, incite a critical visit to MPS concept, in order to reconsider that vertebrates have at least two professional mononuclear phagocyte systems: one derived from monocytes and the other from B-1 cells.

## Supporting Information

Figure S1 **B-1 cell purification strategy.** Peritoneal cells from BALB/c mice were harvested and stained with fluorochrome-labeled antibodies directed against CD19 and CD23 for flow cytometric analysis and cell-sorting. (A) Doublet cells were excluded according to forward scatter profiles (FSC-A×FSC-H). (B) Considering only single cells, the lymphocyte region was determined based on SSC-A×FSC-A parameters. (C) CD19^+^CD23^−^ cells (B-1 cells) were selected from the lymphocyte region.
(TIF)
