# Supplementary figures and images for: Could a B-1 Cell Derived Phagocyte “Be One” of the Peritoneal Macrophages during LPS-Driven Inflammation?
Source: PLoS One. 2012 Mar 30;7(3):e34570. doi: 10.1371/journal.pone.0034570 (PMC3316698; doi:10.1371/journal.pone.0034570)

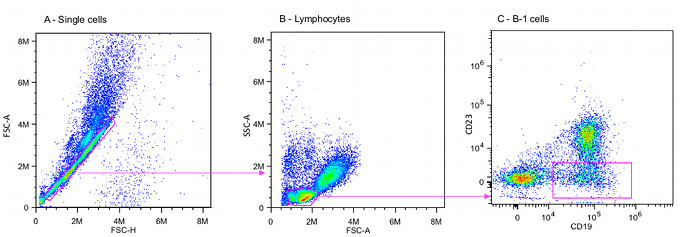

Supplement: Figure S1 — B-1 cell purification strategy. Peritoneal cells from BALB/c mice were harvested and stained with fluorochrome-labeled antibodies directed against CD19 and CD23 for flow cytometric analysis and cell-sorting. (A) Doublet cells were excluded according to forward scatter profiles (FSC-A×FSC-H). (B) Considering only single cells, the lymphocyte region was determined based on SSC-A×FSC-A parameters. (C) CD19+CD23− cells (B-1 cells) were selected from the lymphocyte region. (TIF) [file pone.0034570.s001.tif]
